# The GROWBABY Research Network: A Framework for Advancing Health Equity Through Community Engaged Practice-Based Research

**DOI:** 10.1007/s10995-022-03564-6

**Published:** 2023-01-02

**Authors:** Clare Viglione, Renée Boynton-Jarrett

**Affiliations:** Division of General Pediatrics, Department of Pediatrics, Boston Medical Center, 801 Albany Street, 02119 Boston, MA USA

**Keywords:** Practice-based research, Pragmatic research, Community engagement, Community-based participatory research, Implementation science, Equity

## Abstract

**Purpose:**

Preventive health care, delivered through well child care visits, serves as a universal and primary entry point for promoting child wellbeing, yet children with lower socioeconomic status and children of color receive less consistent and lower quality preventive health care. Currently, limited research exists comparing models for delivering preventive care to children and their impact on longstanding racial/ethnic and socioeconomic inequities.

**Description:**

Practice-based research networks can help to advance health equity by more rapidly studying and scaling innovative, local models of care to reduce racial/ethnic and socioeconomic inequities in primary care and preventive care utilization. This paper outlines a framework of community engagement that can be utilized by practice-based research networks to advance health equity and details the application of the framework using the GROWBABY Research Network (GROup Wellness Visits for BABies and FamilY Research Network).

**Assessment:**

The GROWBABY Research Network launched in 2020, engaged clinical practices utilizing this unique model of group well childcare - CenteringParenting® - with the following goals: to promote collaboration among researchers, clinicians, patients, and community members; facilitate practice-based research; and increase the use of shared assessment measures and protocols. As a research collaborative, the GROWBABY Research Network connects clinical partners facing similar challenges and creates opportunities to draw upon the assets and strengths of the collective to identify solutions to the barriers to research participation.

**Conclusion:**

Primary care, practice-based research networks like the GROWBABY Research Network that intentionally integrate community engagement principles and community-based participatory research methods can advance equitable health care systems and improve child wellbeing.

## Significance Statement

*What is already known?* Practice-based research networks have had a strong history of collaboration at all stages of study development among practitioners, investigators, network administrators, and office staff independent of study topic and more recently an emerging inclusion of patients as research partners (Hickner & Green, 2015).

*What this article adds?* This article extends prior work on community-based participatory research in practice-based research networks by outlining a new participatory framework for advancing health equity through practice-based research networks with specific application and examples from the GROWBABY Research Network. We share explicit equity-centered approaches to community research partnerships, including the development of research infrastructure and systems that share power, measurement of structural racism and community strengths, data sharing and coordination, which together may yield a better understanding of how health inequities are constructed, operationalized, and perpetuated in order to identify the most promising strategies to address them.

## Introduction

Almost half of children in the United States lived in poverty *prior* to the COVID-19 pandemic. COVID-19 has forced an additional 2.5 million children into poverty (Parolin et al., 2020) and approximately 43,000 children have lost a parent during the pandemic (Kidman et al., 2021). Black and Latino children account for more than half of children experiencing poverty in the US (Center KCD, 2019), an inequity that has worsened during the pandemic. Research consistently shows that when children live in low-income households, even for brief periods of time, they are more likely to be in poor health, be hospitalized, at-risk of developmental delays, and experience mental health disorders, obesity, and difficulties learning in school (Beck et al., 2018; Jensen et al., 2017; Radey et al., 2021).

Preventive health care, delivered through well child care visits, serves as a universal and primary entry point for promoting child wellbeing. Yet children with lower socioeconomic status and children of color receive less consistent and lower quality preventive health care (Health UDo, 2018). According to the Centers for Disease Control, the pandemic also led to a sharp decline in children’s engagement in preventive healthcare exacerbating persistent health inequities. Delays in child health visits are associated with delays in vaccinations, higher rates of hospitalizations, emergency department visits, and increased health care costs making public health and healthcare efforts more urgent (Tom et al., 2010; Pittard, 2011; Kheirkhah et al., 2015).

Despite national initiatives charged with closing these gaps, inequities in child health, healthcare access and utilization remain pervasive. Currently, limited research comparing models for delivering preventive care to children, and their impact on critical child health outcomes and longstanding racial/ethnic and socioeconomic inequities exists. We cannot achieve health equity without addressing systemic racism—interconnected policies, institutions, and practices that are rooted in history, culture, norms, and ideologies that maintain and justify inequality (Boynton-Jarrett et al., 2021).

Practice-based research networks (PBRNs) can support research, innovation, and interventions to reduce racial/ethnic and socioeconomic inequities in primary care and preventive care utilization (Hickner & Green, 2015). PBRNs are effective for building a sustainable evidence-base for primary care and are grounded in community engagement and community-based participatory research (CBPR) practices (Westfall et al., 2006; Westfall et al., 2009). We acknowledge and build on the work of Williams and colleagues’ application of CBPR to PBRNs (Williams et al., 2009) through explicit consideration of the impact of participatory processes that intentionally center equity in the approach to community partnership and stakeholder engagement, develop research systems that share power, and improve measurement of structural racism and community strengths in efforts to promote health equity. In this paper, we describe the development of the GROWBABY Research Network (GROup Wellness Visits for BABies and FamilY Research Network) and outline a framework of key process elements for equitable community partnership and stakeholder engagement that can be utilized by PBRNs to advance health equity. We crosswalk specific examples of how the GROWBABY Network applies framework components to its structure and function.

## Description

The GROWBABY Research Network (GROup Wellness Visits for BABies and FamilY Research Network) launched in 2020 with the following goals: to promote collaboration among researchers, clinicians, patients, and community members; facilitate practice-based research; and increase the use of shared assessment measures and protocols. As a research collaborative, GROWBABY connects clinical partners facing similar challenges and creates opportunities to draw upon the assets and strengths of the collective to identify solutions to the barriers to research participation. GROWBABY operates as a community-based PBRN and is an initiative of the CRADLE (Childhood Research to Advance Developmental-Health Learning and Equity) Lab, the research arm of the Vital Village Networks organization at Boston Medical Center which functions as GROWBABY’s backbone organization.

Vital Village Networks’ mission is to develop community-based strategies to promote child wellbeing and advance health and educational equity through research, data sharing, and collective action. Developing networks and cross-sector collaborations where community residents co-lead and co-design is a central strategy. Vital Village Networks CRADLE Lab staff use a community-based participatory research approach and an equity-centered collective impact framework, incorporating process elements like a common agenda, continuous communication, and backbone organizational support, for meeting structure and operations to facilitate the development of the GROWBABY Research Network (Sandel et al., 2016; Kania et al., 2022).

The GROWBABY Steering Committee includes 11 members representing 7 partner sites (3 Federally Qualified Health Centers (FQHCs) and 4 Academic Centers). The Steering Committee meets monthly via Zoom to discuss GROWBABY initiatives and collaboratively develops shared agreements, such as Memorandums of Understanding, Data Sharing Agreements, and determines research priorities. The Steering Committee has met 16 times since the inception with additional, ad-hoc small group meetings. See Table 1 for Steering Committee Member characteristics.

**Table 1:** GROWBABY Network Steering Committee Memers

Member	State / City	Website	# Steering Committee Members	Organization Type Academic Medical Center (AMC) Federally Qualified Health Center (FQHC)	# Practice sites	# Sites with group well child care	# Providers trained in group care	# Patients served annually
El Rio Health	Arizona / Tucson	elrio.org	2	FQHC	15	1	3	112,765
Tower Health - Clildren’s Health Center	Pennsylvania / West Reading	towerhealth.org	2	AMC	1	1	3	17,000
Covenant Community Care - Detroit	Michigan / Detroit	covenantcommunitycare.org	2	FQHC	6	1	3	17,309
Einstein Medical Center Philadelphia - Pediatric and Adolescent Ambulatory Center	Pennsylvania / Philadelphia	einstein.edu	2	AMC	1	1	40	20,000
UM BWMG - Pediatrics at Glen Burnie	Maryland / Glen Burnie	umms.org/bwmc	2	AMC	2	2	12	1,200
CommUnity Care - East Austin	Texas / Austin	communitycaretx.org/locations/	1	FQHC	30	1	3	8,500
UCSF Department of Family and Community Medicine*	San Francisco / California	fcm.ucsf.edu/	1	AMC				N/A

*UCSF serves as a research partner providing research input and expertise, not a clinical partner.

Vital Village Networks’ CRADLE Lab, the backbone organization of GROWBABY, engages with dozens of childhood practitioners across the country who are working hard to meet the social and psychological needs of the families they serve, in addition to providing routine medical care. Frequently from under-resourced clinics or FQHCs, practitioners often work overtime to spearhead clinical programs that hold promise of improving breastfeeding rates, child neurodevelopment and language, and physical and mental health. Despite barriers to engagement in academic research and traditional funding streams (e.g., time, research and publication costs, and data analytic requirements) clinical sites have responded to immediate, localized needs and developed culturally sensitive interventions unique to their context and clinic. Community-based providers, Promotores, Community Health Workers and other providers are curating unique models of care and generating data and observing results firsthand. For instance, listening to patients’ stories encouraged a practitioner of Reading Children’s Health Center to tailor group care for opioid addiction and similarly, a facilitator of group care at BayClinic UM Baltimore Washington developed a model of group well childcare focused on maternal depression.

The GROWBABY Research Network partners with these FQHCs to provide opportunities to participate in research not routinely offered to community-based clinics. FQHCs serve a greater proportion of low-income, uninsured, and publicly insured patients, yet FQHCs are frequently excluded from the research enterprise (Hacker et al., 2013). The way research is currently funded, large academic centers connected to robust healthcare systems receive the majority of federal funding. Yet, models that hold promise in lifting children out of poverty - children living in under resourced communities of color where medical mistrust and lack of partnership opportunities has reduced participation in research, often live outside of academia and in community settings (Bloomfield & Rising., 2013). We also know from studying care models such as the CenteringParenting® model of group well childcare, promising and effective models do exist that equip families with tools and social support to buffer children from adversity, and prevent poor mental, physical health outcomes over the lifecourse (Bloomfield & Rising., 2013). For example, CenteringParenting®, brings 6–8 parents and their same-age infants together in community with their providers to foster a safe environment for new parents to learn from each other, ask uncomfortable questions, and forge lasting bonds. Sites report promising preliminary data demonstrating CenteringParenting is associated with improved immunization rates, visit attendance, and enhanced patient and provider satisfaction (Irigoyen et al., 2021; Gullett et al., 2019; Jones et al., 2018). The GROWBABY Research Network allows us to expand upon preliminary work and conduct multi-site research to investigate models on a broader scale. GROWBABY has two pilot studies, referenced in this manuscript, that have been approved by the Boston University Medical Campus Institutional Review Board.

### Assessment

Given the severity of the pandemic on American families, structural changes are needed to improve health equity and enhance wellbeing for underserved American children. A potential solution is a community-driven, PBRN which intentionally employs participatory practices into its development and expansion. Community-based participatory research enhances the feasibility, relevance and cultural appropriateness of research outcomes, and facilitates translation of research to practice (Kwon et al., 2018). PBRNs are effective for building a sustainable evidence-base for primary care and are grounded in community engagement and community-based participatory research (Westfall et al., 2006). By co-locating researchers, practitioners, and community members, PBRNs can cultivate effective and sustainable partnerships with stakeholders to ultimately address longstanding inequities in our healthcare system.

#### Community-Driven Practice-Based Research Sets a New Agenda to Advance Equity in Primary Care

**Fig. 1:**
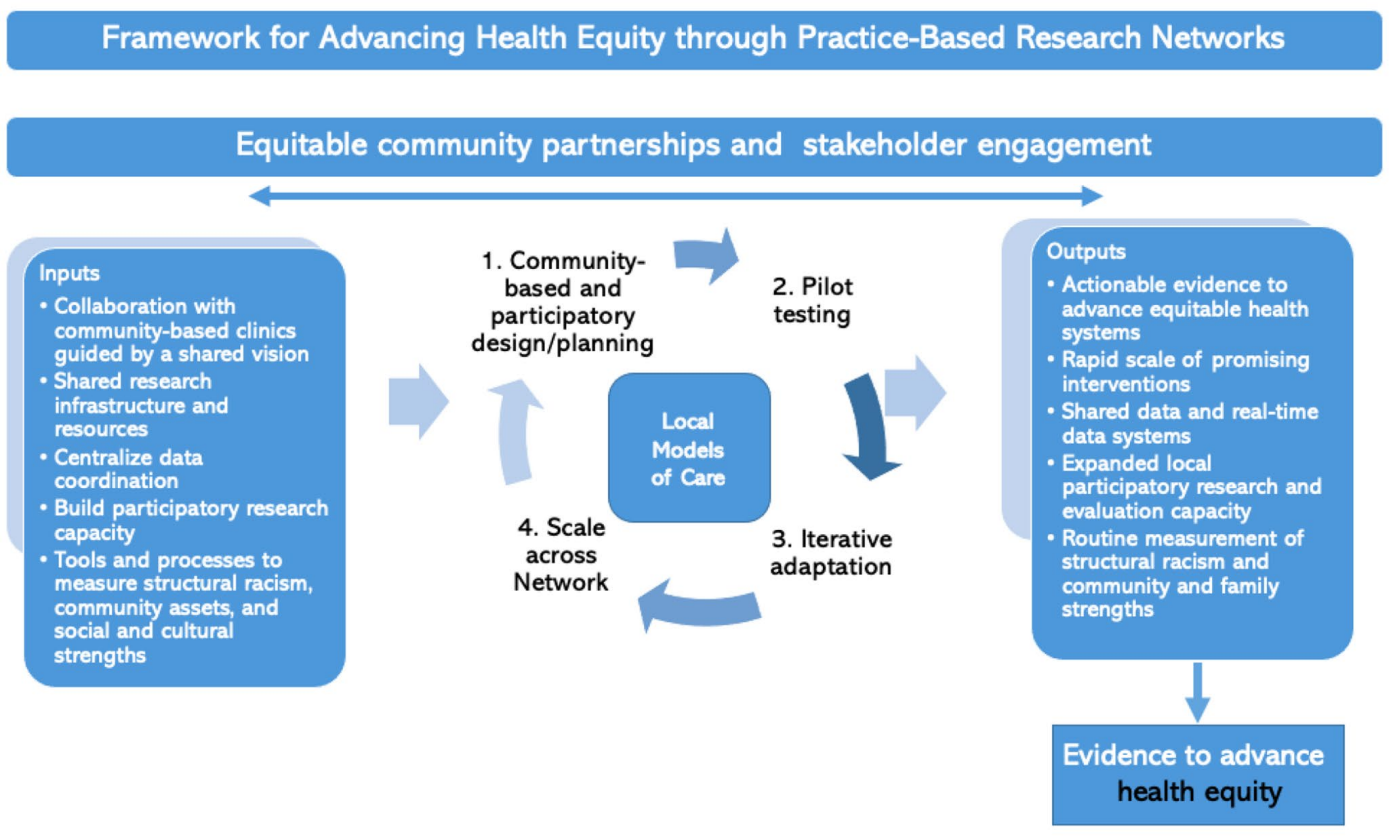
Figure 1 displays a working framework to illustrate how Practice-Based Research Networks grounded in community engagement practices might advance health equity

First, any equity effort requires community-driven solutions. To promote equitable healthcare systems that cater to diverse cultural and geographic needs, elevating locally adapted models of clinical care, such as group pediatric care, is a significant strategy. Community-based, culturally adapted, group care models cannot be manufactured by researchers outside of the local context, and a PBRN focused on extant programs allows us to study localized innovation while systematically and pragmatically sharing learnings across sites by leveraging the power of centralized data sharing. PBRNs focused on pragmatic and agile innovation allow us to more rigorously evaluate community-driven, culturally adapted and localized models of care to move the needle towards equity (Hekler et al., 2016). Figure 1 displays our framework for advancing health equity through PBRNs. The framework is an expanded logic model with inputs (e.g., collaboration with community-based clinics), activities (e.g., iterative pilot testing modeled after Plan-Do-Study-Act cycles), outputs (e.g., rapid scale of promising interventions) and outcomes (i.e., advance health equity). Importantly, these central inputs work to address the drivers of inequities by intentionally and systematically focusing on equitable collaboration with community partners, sharing power and research infrastructure, building community research capacity, and developing methods and measures to address structural racism (Boynton-Jarrett et al., 2021). We hypothesize that applying these elements in practice will work synergistically to enable the advancement of health equity. Foundational to the framework is ‘equitable community partnership and stakeholder engagement.’ Although many stakeholder engagement frameworks exist, we are not aware of any published frameworks with an explicit linkage to equity through the application of participatory and anti-racist practices within PBRNs.

Second, PBRNs are rooted in principles of stakeholder engagement to enhance the relevance of research questions and support the translation of evidence-based research into sustainable community change. A model that both recognizes and emphasizes the strengths of people and communities (including the clinician community) to work collaboratively to bring about positive change and their capacity for problem solving (Williams et al., 2009). Similarly, Citizen Science is “the collection and analysis of data relating to the natural world by members of the public, typically as part of a collaborative project with professional scientists” (Bonney et al., 2016). In line with Citizen Science, a PBRN brings capacity building resources including training, technical assistance, and research knowledge to smaller community and rural clinics and their stakeholders to democratize the research process. The GROWBABY Research Network centers equity when using a collective impact model to guide research partnership, collaboration and meeting structure with a common agenda, continuous communication, and backbone organizational support that begins with listening to the community, addressing power imbalances in research partnerships, creating shared governance and decision-making processes, and advancing sustainable community-leadership for research (Sandel et al., 2016; Kania et al., 2022).

Third, smaller clinics are often excluded from the research conversation. Smaller clinics based in community settings make up the majority of care for low-income populations. Many of these lower-resource and smaller clinics operate on siloed electronic medical records (EMRs) of which funders and researchers sometimes avoid given the complexities with linking data management systems with more dated or homegrown EMRs. However, current evidence collected in academic health services may not generalize to lower resource settings because of exclusion of low-income service settings and BIPOC (Black, Indigenous, and people of color) individuals from clinical trials (Hacker et al., 2013; Nazha et al., 2019). It is critical that we include smaller health clinics in the research process if we ultimately need to scale-out models across lower-resource and community settings. Further community health centers and FQHCs face routine and predictable barriers in translation of evidence-based practices including staff engagement, space, funding, technology, and cultural and translation barriers (Gagnon et al., 2022; Fam & Ferrant, 2018; Wakida et al., 2018; Chartrand et al., 2019; Brady et al., 2020; Toscos et al., 2018). A PBRN allows us to share resources and insights and leverage the power and capital of multiple clinical partners of varying size to address these challenges, while soliciting external researchers and funders to partner with us for study (DeVoe et al., 2012).

Further, shared metrics across sites will facilitate a robust evaluation (Surbhi et al., 2020). Within a PBRN, we can work together to identify and agree upon shared metrics to evaluate success of our programs. We can work with research network partners to build consensus on what “success” looks like and identify feasible, appropriate, scalable outcome indicators such as life course outcomes, academic outcomes, stress reactivity, resiliency, and linkages with social resources in addition to important implementation outcomes such as reach, adoption, and fidelity.

**Fig. 2:**
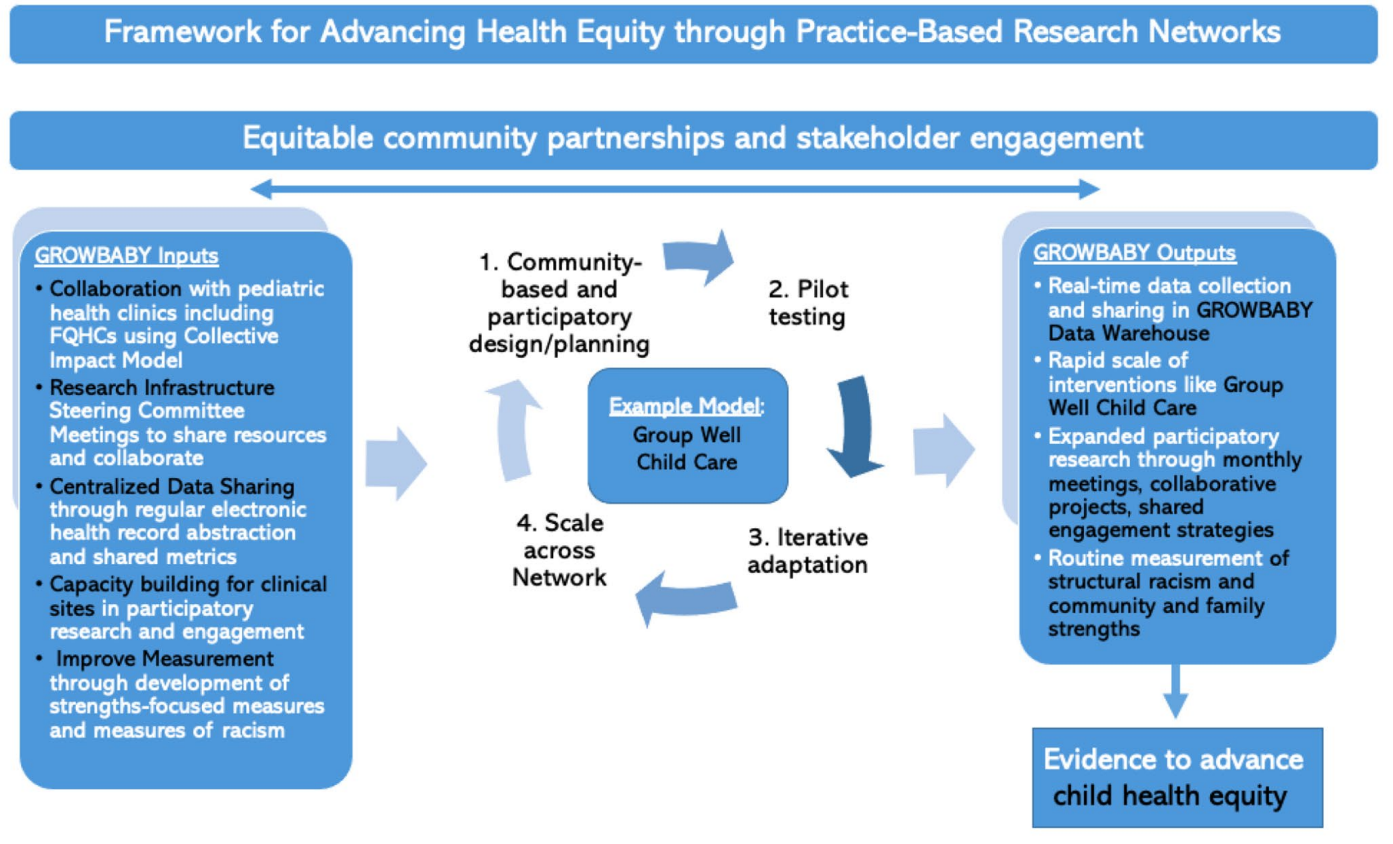
This Figure 2 overlays specific and applied examples from the GROWBABY Research Network onto the framework (see Fig. 1) to more clearly articulate how Practice-Based Research Networks grounded in community engagement and anti-racist practices might advance health equity

A research network allows us to optimize our data collection pathways (Van Weel, 2005), integrate streams of data from many clinical sites, and share historical datasets. With that, larger datasets allow us to answer more questions, more quickly, with more precision, and boost reliability when advocating for strategic policy changes. As an example, the GROWBABY Network is currently collaborating on multi-site data extraction of several indicators of child-wellbeing from existing electronic charts (e.g., preventive visit attendance, breastfeeding rates, and immunization timeliness). See Fig. 2 which cross-walks specific and applied examples from the GROWBABY Research Network onto the aforementioned framework domains.

Lastly, PBRNs will likely accelerate the generation of research results. The COVID-19 pandemic has dramatically increased poverty nationally and disproportionately among Black and Latino children, requiring us to think differently and act now. In the traditional academic paradigm, it takes *17 years* to translate clinical research into routine practice (Westfall et al., 2007). Given that pediatric research makes up a mere fraction (12%) of total NIH research (Stoll & Taegtmeyer, 2018), we cannot afford to wait for interventions targeting underserved children to be designed, piloted, tested, re-tested, implemented and disseminated to help us cope with adversities facing American families today. To accelerate progress, we can utilize the PBRN infrastructure to work in partnership with providers and communities serving children and families on the frontlines and scale interventions. PBRNs can fuel the development of multi-site, large population trials to increase the pace of data collection, interpretation, and innovation testing and optimization.

## Conclusion

If we want equitable health care systems that can meet the urgent needs of American families, ambitious change will be required. Prioritizing research networks, infrastructure, design and approaches that are participatory and center equity and improve measurement of community strengths and structural racism may unearth new solutions and evidence. True research partnerships with clinical stakeholders from under-resourced contexts and community members most impacted by inequities are not only ethical, but also enable the development of interventions and implementation strategies that are equitable and effective. Primary care-focused, PBRNs like the GROWBABY Research Network built on an explicit framework of community engagement can advance equitable health care systems and support child wellbeing.

## Data Availability

We can share any of the information upon request at clare.viglione@bmc.org.
